# Vaccination and Small Pox

**Published:** 1872-01

**Authors:** 


### Vaccination and Small Pox.

It is noticeable that though Jenner discovered the protective power of vaccination, still small pox epidemics prevail in our large cities and produce most alarming and fatal effects. There is no doubt that vaccination and *re*-vaccination is a complete protection from small pox. Still the question is regarded by the public as quite unsettled, and the inquiry is made, if so, why does the disease prevail so extensively, and why is varioloid so common in those who have been vaccinated? The question should be answered to the public and made plain so far as may be; but the impossibility of teaching medical facts, to the unprofessional, is very well understood.

The faults of our present system of protection from small pox are still very numerous; they scarcely require enumeration to medical men.

1st. There is not attached to the event of being protected from small pox, anything of the importance which it really demands. It is regarded as a matter of almost no consequence at all, to be attended to when some of the neighbors are announced as having the disease; whereas, it should be looked upon as one of the principal events of life, and be recorded with birth, baptism, marriage and death.

2nd. *Re*-vaccination should be understood to be essential to protection—protection from varioloid or modified small pox. In other words, parents should know that their children are not *protected* in any true, popular sense, until pure vacine virus will not produce *its* characteristic effects, and that there is every reason to believe when *thus* vaccinuated they are more perfectly protected than are those who have had small pox, from future attacks.

3d. Better ideas should prevail both with the profession and public as to what *is* a real vaccine vesicle, and how it can only be produced by the vaccine virus; that eczematous scales, scales from second vaccination and the second scale from true vaccine sore are incapable of producing the *vaccine vesicle*, or of protecting at all, from small pox.

When these simple facts are appreciated—when the event of being vaccinated is regarded in its true light, and when our present plan of gratuitous, unwatched, promiscuous, uncared for, and almost wholly useless plan of public vaccination is superceded by an intelligent, carefully watched, thoroughly executed procedure of *protection*, such as is within certain and easy reach, then no more small pox. That it prevails in Buffalo and other cities is proof of ignorance and poor government and criminal neglect.

